# Insulin and LiCl Synergistically Rescue Myogenic Differentiation of *FoxO1* Over-Expressed Myoblasts

**DOI:** 10.1371/journal.pone.0088450

**Published:** 2014-02-13

**Authors:** Yi Ju Wu, Yen Hsin Fang, Hsiang Cheng Chi, Li Chiung Chang, Shih Ying Chung, Wei Chieh Huang, Xiao Wen Wang, Kuan Wei Lee, Shen Liang Chen

**Affiliations:** Department of Life Sciences, National Central University, Jhongli, Taiwan; University of Minnesota Medical School, United States of America

## Abstract

Most recent studies reported that FoxO1 transcription factor was a negative regulator of myogenesis under serum withdrawal condition, a situation not actually found *in vivo*. Therefore, the role of FoxO1 in myogenesis should be re-examined under more physiologically relevant conditions. Here we found that FoxO1 was preferentially localized to nucleus in proliferating (PMB) and confluent myoblasts (CMB) and its nuclear exclusion was a prerequisite for formation of multinucleated myotubes (MT). The nuclear shuttling of FoxO1 in PMB could be prevented by leptomycin B and we further found that cytoplasmic accumulation of FoxO1 in myotubes was caused by the blockade of its nuclear import. Although over-expression of wildtype *FoxO1* in C2C12 myoblasts significantly blocked their myogenic differentiation under serum withdrawal condition, application of insulin and LiCl, an activator of Wnt signaling pathway, to these cells successfully rescued their myogenic differentiation and generated myotubes with larger diameters. Interestingly, insulin treatment significantly reduced FoxO1 level and also delayed nuclear re-accumulation of FoxO1 triggered by mitogen deprivation. We further found that FoxO1 directly repressed the promoter activity of myogenic genes and this repression can be relieved by insulin and LiCl treatment. These results suggest that FoxO1 inhibits myogenesis in serum withdrawal condition but turns into a hypertrophy potentiator when other myogenic signals, such as Wnt and insulin, are available.

## Introduction

Transcription factors of the FoxO family, including FoxO1 (FKHR), FoxO3 (FKHRL1), FoxO4 (AFX), and FoxO6, have been discovered to play important roles in a diverse sets of cellular physiological functions [1]–[3]. They can function by direct binding to DNA or by tethered to the target site through protein-protein interaction with other transcription factors, such as nuclear receptors and HNF4, [4]. Their transcriptional modulating function is critically regulated by signaling pathways, which act by post-translational modifications of FoxO proteins on specific residues [5]. For instance, signals transduced by binding of insulin to the plasma membrane receptor activates PI3k-Akt pathway, which leads to the phosphoryaltion of T24, S256 and S319 residues in FoxO1. Phosphorylated FoxO1 tends to be shuttled out of nucleus and thus lost their binding to target regulatory elements [6], [7]. These 3 Akt targeted sites, named as T1, S1, and S2, are conserved from *Daf16* in *C. elegan*s to its orthologs in mammals [8]. In addition to these Akt-targeted sites, multiple residues in FoxO1 are also phosphoryalted by other kinases, including CDK2, DYRK1 and CK1 [9]–[11].

Over-expression of FoxOs in various cell types has demonstrated that they can induce cell cycle arrest at G1-S checkpoint through activation of *p27^Kip1^* or at G2-M checkpoint by activation of *GAD45* [6], [12]. Mutation of these cell cycle regulatory transcription factors has been a recurrent target in the rhabdomyosarcoma, an aggressive malignant muscle tumor type that account for 5–8% of all cases of childhood cancer [13]. Chromosome translocation in RMS has created chimeric genes made of *PAX3/PAX7* and *FKHR*. Both PAX3-FKHR and PAX7-FKHR have the ability to induce malignant muscle phenotype when induced into non-muscle lineage [14], [15]. They have potent transforming effects and are strong inhibitors of myogenic differentiation, implying that normal function of FoxO1 might be important for myogenesis.

Muscle precursor cells are derived from somitic cells and they migrate out to specific sites in the embryo to fuse into multinucleated mature myocytes [16]. The myogenic lineage is determined by the expression of either *MyoD* or *Myf5* in myogenic stem cells to generate proliferating myoblasts. Upon differentiating stimuli, the expression of *Myogenin, Mrf4,* and *Mef2c* in myoblasts is induced to facilitate the execution of the myogenic program, including cell cycle exit, expression of contractile proteins, and fusion of myoblasts into multinucleated myotubes [17]. The fusion of myoblasts is a poorly understood process that can be affected by signals released from extracellular matrix, cell-cell adhesive molecules, paracrine factors and even community effects [18]. Since FoxOs are abundantly expressed in mature skeletal muscle and its stem/progenitor cells [19], it is interesting to know what functions do FoxOs play in myogenesis.

Previous studies had discovered that ectopic over-expression of constitutively active F*oxO* mutants (with their Akt target sites mutated into alanine) in myoblasts could either induce atrophy or inhibit differentiation [20]–[22]. Transgenic mice over-expressing *FoxO1* showed reduced muscle mass, down-regulated type I fiber genes and impaired glycemic control [23]. It has been demonstrated that FoxOs induce muscular atrophy through activating the transcription of muscle-specific ubiquitin ligases *Atrogin-1* and *MuRF1* as well as a novel ubiquitin-binding protein referred to as ZNF216 [20], [21], [24]. However, other groups have observed enhanced myogenesis when FoxO1 or FoxO3 was expressed in mouse primary myobalsts and C2C12 myoblasts [25], [26]. These conflicting observations suggest that FoxOs might have dual roles in myogenesis and further investigations are required to clarify their roles in this process.

To address this issue, we over-expressed *FoxO1* in C2C12 myoblasts and carefully observed their subcellular localization during myogenesis. Under serum withdrawal condition, both morphological and molecular evidences shown that over-expression of *FoxO1* in C2C12 myoblasts significantly blocked their myogenic differentiation. However, application of insulin and LiCl to C2C12-*FoxO1* cells successfully rescued their myogenic differentiation and generated myotubes with larger diameters. Stage-specific shuttling of FoxO1 between nucleus and cytoplasm was observed and this could be altered by treatment with insulin. We further found that FoxO1 directly repressed the promoter activity of myogenic genes and this repression can be relieved by LiCl and insulin treatment. These results suggest that FoxO1 inhibits myogenesis under the condition of serum withdrawal but turns into a hypertrophy potentiator when other myogenic signals are available.

## Materials and Methods

### Plasmids

The coding sequence (CDS) of *FoxO1* were released from parental vector, pCDNA3-GFP-*FoxO1* (a generous gift from Dr. William Seller, Dana-Faber Cancer Institute, Boston, MA), by *BamH*I and *Xba*I digestion before blunted by Klenown reaction. Then, it was inserted into the blunted *Xho*I site of pMSCV-neoEB vector for generating retrovirus. The expression vectors for *MyoD*, *Myogenin*, *Myf-5*, *MRF-4* and *Mef2C* were described previously [27]. *Mef2c* promoter (−3355∼+89) was amplified from mouse DNA and inserted into the *EcoR*I (blunted) and *BamH*I sites of pGL3 basic vector. The *Myogenin* and *M-cadherin* promoters have been described in our previous works [28], [29], and the *MCK* promoter is a generous gift from Dr. Eric Olson at University of Texas Southwestern [30].

### Cell Culture and Establishment of Stable Clones Over-expressing FoxO1

C2C12 myoblasts were kept in growth medium (GM, DMEM supplemented with 20% FCS and 110 mg/L sodium pyruvate) and split every 2–3 days to prevent contact of cells. To make infectious retrovirus, pMSEV-neoEB vectors [31] carrying *FoxO1*-wt or –AAA CDS was transfected into GP+E-86 cells, a retrovirus package cell line [32], overnight and started selection with G418 (400 µg/ml) 48 hr after transfection. After confirming the expression of ectopotic *FoxO1*with RT-PCR, retrovirus was harvested from the culture medium of GP+E-86 cells and transferred to the medium of C2C12 for infection. Infection was allowed to proceed for 2days and then G418 (400 µg/ml) was added and the selection was continued for 2–3 weeks. The expression level of *FoxO1* in C2C12 stable clones was confirmed at both RNA and protein levels. The expression level of FoxO1 was stable during the experimental period (at least 20 passages). Both GP+E-86 cells and pMSCV-neoEB vector are generous gifts from Dr. Robert G. Hawley (The George Washington University Medical Center, Washington, DC 20037). For terminal differentiation assays, parental and stable clone C2C12 myoblasts were allowed to grow confluent and then medium was replaced with differentiation medium (**DM**, DMEM containing 2% horse serum). Cells were allowed to differentiate for 96 hr before harvested for isolating total RNA or immunocytochemical staining of MHC.

### Quantitative Real-time RT-PCR

The protocol for real-time PCR has been described before [27]. Briefly, total RNA was extracted from the C2C12 and Sol8 myoblasts using TRIZOLE (Life Technology; Rockville, MD) according to the supplier’s instruction. Then, the first strand of cDNA was synthesized using the Superscript II kit (Life Technology; Rockville, MD). Real time PCR was performed in a 25 µl reaction mixture containing 5 µM forward/reverse primers, 1X SYBR Green reaction mix (Applied Biosystem; Werrington, UK), and various amounts of template. Different amounts of template were used in the same reaction to make sure the linear amplification of PCR products. *Gapdh* was used as internal control amplified in the same PCR assay. The primer sets used for quantification of myogenic gene expression are listed as in table 1. All reactions were performed in ABI 7300 sequence detection system.

**Table 1:** The sequences and amplicon sizes of the primer sets used in this study.

Gene	Amplicon size	Primer sequence
*MyoD*	204 bp	FP: 5′-ggg tac gac acc gcc tac ta-3′
		RP: 5′-gtt ctg tgt cgc tta ggg at-3′
*Myogenin*	166 bp	FP: 5′-cca gtg aat gca act ccc aca gc-3′
		RP: 5′-aga cat atc ctc cac cgt ga-3′
*Myf-5*	132 bp	FP: 5′-cct gtc tgg tcc cga aag aac-3′
		RP: 5′-tag acg tga tcc gat cca caa t-3′
*Mrf4*	190 bp	FP: 5′-gca ccg gct gga tca gca aga g-3′
		RP: 5′-ctg agg cat cca cgt ttg ctc c-3′
*Mef2c*	175 bp	FP: 5′-gat ggg cg gaga tct gac a-3′
		RP: 5′-gaa cgc gga gat ctg gct tac-3′
*Atrogin-1*	149 bp	FP: 5′-cag cct gaa cta cga cgt c-3′
		RP: 5′-gct tcc ccc aaa gta cag ta-3′
*p21^Cip1^*	135 bp	FP: 5′-gcc gaa aac gga ggc aga c-3′
		RP: 5′-aag atg ggg aag agg cct cct ga-3′
*MHC*	103 bp	FP: 5′-tgc caa ggg cct gaa tga-3′
		RP: 5′-gct tcc acc taa agg gct gtt-3′
*m36b4*	73 bp	FP: 5′-ggc agc att tat aac cct gaa gtg-3′
		RP: 5′-cgg aca ccc tcc aga aag c-3′
*Gapdh*	190 bp	FP: 5′-cct ctg gaa agc tgt ggc gt-3′
		RP: 5′-ttg gca ggt ttc tcc agg cg-3′
*FoxO1*	97 bp	FP: 5′-tcc cac aca gtg tca aga cta caa-3′
		RP: 5′-ctg ctg tca gac aat ctg aag ga-3′

FP: forward primer.

RP: reverse primer.

### Western Blots

The protocol of western blot has been described before [33]. Briefly, Aliquots of total lysate (100 µg) in RIPA buffer were run on 8% SDS-PAGE gels before blotted onto PVDF membrane. Then, PVDF membranes were extensively washed with 1X PBS containing 0.5% Tween 20 (PBST) before blocked by blocking solution (5% BSA in PBST) for an hour. Both primary and HRP-conjugated secondary antibodies were diluted 1∶1000 in blocking solution and incubated sequentially with the blot. After extensive washes with PBST, the signals was detected by a chemilminescence kit (Amersham Pharmacia Biotech) and visualized on X-ray films (Super RX, Fuji Medical X-film; Tokyo, Japan). For detection of internal control, all the blots were stripped and washed thoroughly in PBST, then, blocked and incubated with Gapdh or Lamin B1 antibody as described above. The extraction of nuclear protein has been described before [34]. Polyclonal and monoclonal FoxO1 antibodies were purchased from Cell Signaling Technology (#9462 and # 2880 respectively). Other antibodies used in this study include anti-β-catenin (MAB1329, R&D systems), anti-Mef2 (SC-133, Santa Cruz), anti-MyoD (554130, BD Pharmingen), anti-Myogenin (556358, BD Pharmingen), Lamin B1 (ab16048, ABcam).

### Immunocytochemistry

After in DM for 96 hr, stable clone cells were washed with cold PBS before fixed in 4% paraformaldehyde for 20 min. Then, they were quenched in 50 mM NH4Cl for 15 min before permeablized in 2% TritomX 100 over night. Cells were incubated in blocking solution (2% BSA and 2% goat serum in PBS) for 20 min followed by incubating with MHC antibody (1: 1000 dilution; clone MY-32, Sigma) over night. After extensive wash with PBS, HRP-conjugated secondary antibody (Goat anti mouse IgG, Santa Cruze) was added and incubated for an hour. The expression of MHC was visualized with AEC substrate kit (Zymed Laboratories) and the cells were counter-stained with hematoxylin and eosin.

### Immunofluorescence

For immunofluorescence analysis, control and *FoxO1* over-expressed C2C12 cells were grown on cover slides held in 12 wells dishes. Cell were washed 3 times with PBS and then fixed in 100% methanol at room temperature for 10 min or in 4% paraformaldehyde for 15 min. After fixation, cells were washed 3 times with PBS and then quenched in PBS containing 50 mM NH_4_Cl to avoid the deleterious effect of the methanol on the antibodies. Cells were then incubated in blocking solution (2% goat serum and 2% BSA diluted in PBS) at room temperature for 30 min before incubated with FoxO1 antibody (#9462, Cell Signaling Technology; diluted 1∶200 in blocking solution) at 4°C for at least 16 hr. Then cells were washed 3 times with PBS before incubated with Alexa Fluor 488 conjugated secondary antibody (A11008, Molecular Probes; diluted 1∶200 in blocking solution) at room temperature for 2–3 hr and then washed 4 times with PBS. To visualize the nuclei, cells were incubated with DAPI (1: 5000 dilution in PBS) at room temperature for 10 min after the secondary antibody incubation and were washed with PBS thoroughly afterward. All the images were observed and photographed under the Carl Zeiss Axio Observer A1 fluorescence microscope with AxioVision software.

### Transient Transfection Assays

The protocol of transient promoter activity assay has been described in our previous works [27]. Briefly, C2C12 cells were split and plated into 12-well culture dishes one day before transfection. Transient transfection assays were performed by adding plasmid DNAs in 50 µl Hepes buffer first and then, Lipofectamin (Life technology, Rockville, MD) in 50 µl Hepes buffer was added to the DNA solution and incubated at room temperature for 15–30 min allowing the DNA/liposome complex to form. Aliquots of culture medium were added to each tube and mixed gently. Medium containing the DNA/liposome complex was transferred to cells. The transfection solution was left overnight before the media was replaced with differentiation medium containing 2% horse serum. Cells were harvested and assayed for luciferase activity 16–24 hr after transfection in a Clarity 2 luminometer (BioTEK; Winooski, VM). All experiments were performed in triplicates and repeated at least 3 times.

## Results

### Effects of FoxO1 Over-expression on Myogenic Differentiation

**Figure 1:**
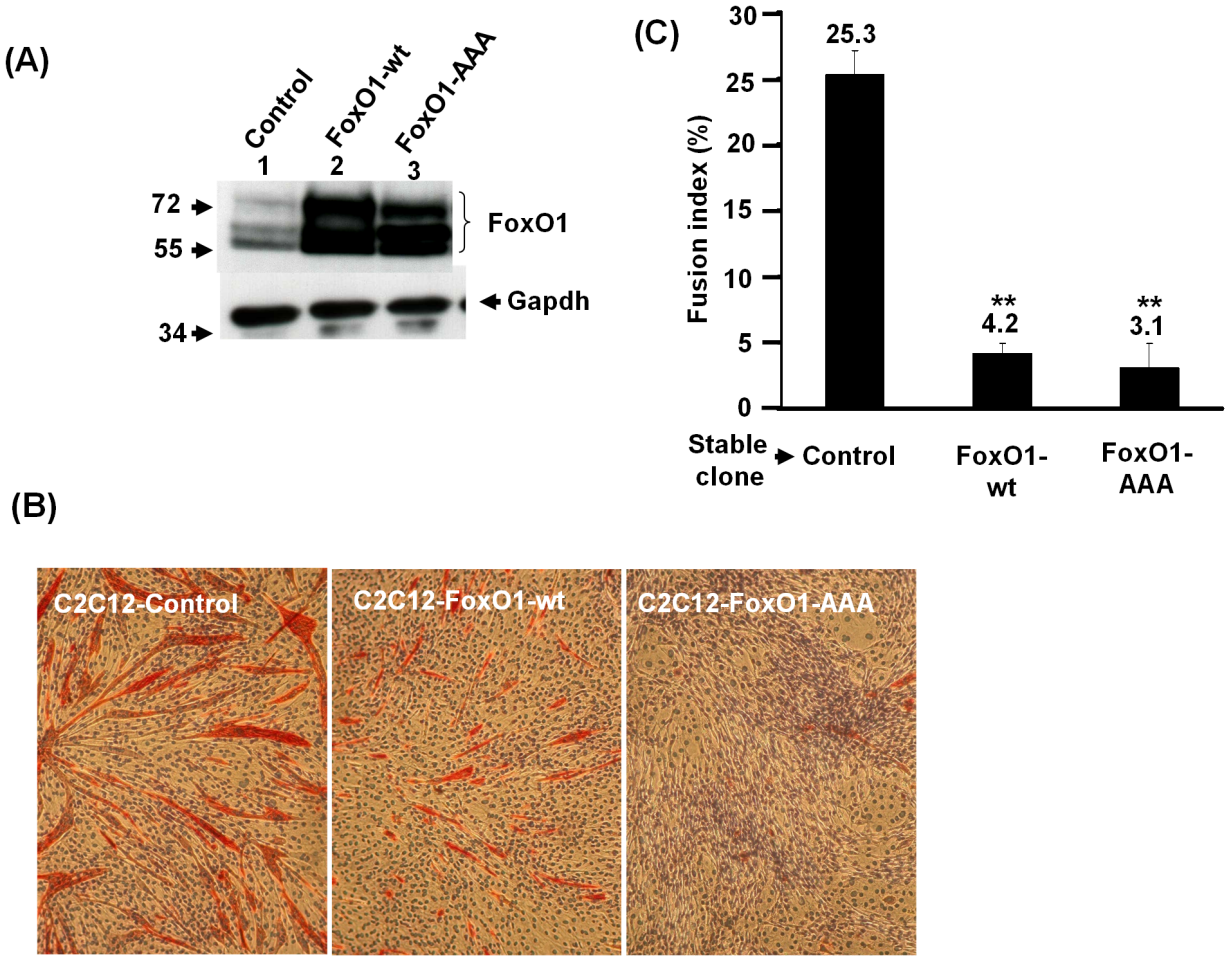
Establishment of *FoxO1* over-expressed C2C12 stable clones. Expression levels of FoxO1 protein in control and C2C12-*FoxO1* stable clones were detected by Western blot. The antibody recognizes both native (bottom band) and phosphorylated (top 2 bands) form of FoxO1. The signal of Gapdh serves as protein inputs control. Protein markers in kilodalton (Kd) are shown to the left in (A). wt: wildtype; AAA: constitutively active mutant. (**B**) and (**C**) confluent *FoxO1* over-expressed C2C12 stable clones were induced to differentiate by changing to differentiation medium (DM) and harvested 96 hr later for immunohistochemical staining of myosin heavy chain (MHC, red). After counter-stained with hematoxylin, the morphology of these stable clones was photographed and shown in (B). The original magnification was 100X. (C), the fusion index (percentage of nuclei in MHC-positive myotubes) of stable clones was calculated in at least 3–5 representing fields and the differentiation assay was repeated at least twice. **: p<0.01 as compared with that of control cells.

To examine the function of FoxO1 in myogenic differentiation, we started with the over-expression of *FoxO1* in C2C12 myoblasts by infecting them with retrovirus carrying wildtype (wt) or constitutively active (AAA, in which the 3 Akt sites were mutated to alanine) form of *FoxO1* coding sequence and selected with antibiotics (G418) for 2–3 weeks to generate stable clones (C2C12-FoxO1) expressing *FoxO1*. The over-expression of *FoxO1* in stable clones was confirmed by Western blot and the FoxO1 shown up as 3 bands due to the recognition of both phosphorylated and native FoxO1 by the antibody used (Fig. 1A). To properly represent the original C2C12 population, both control and C2C12-FoxO1 cells used in this study were polyclonal.

The effect of *FoxO1* over-expression on C2C12 moygenic differentiation was tested by serum withdrawal induced myogenic differentiation, in which both control and C2C12-FoxO1 cells grown to confluent were induced to differentiate by changing to differentiation medium (DM) containing 2% horse serum. Morphologically, over-expression of both wildtype and active forms of *FoxO1* retarded the formation of multinucleated myotubes (Fig. 1B) and the expression of myosin heavy chain (MHC). Calculation of nuclei in the MHC-expressing myotubes (Fig. 1C) shown that fusion index (nuclei in myotubes/total nuclei) was significantly reduced in C2C12-FoxO1-wt and -AAA cells (4.2% and 3.1%, respectively) as compared to that of control cells (25%).

### Subcellular Localization of FoxO1 during Myogenic Differentiation

**Figure 2:**
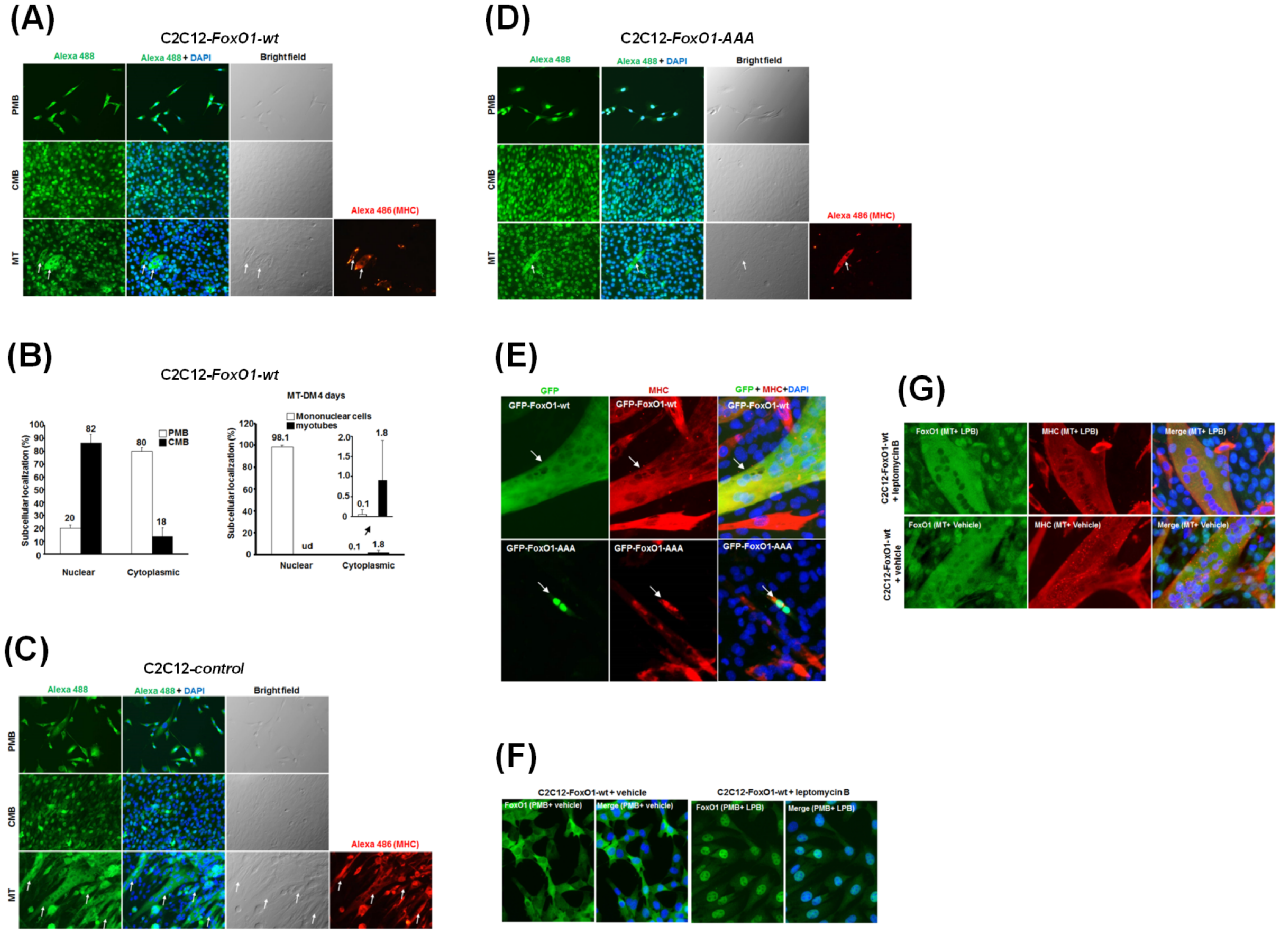
FoxO1 shuttles between nucleus and cytoplasm during myogenic differentiation. The subcellular localization of FoxO1 in C2C12-FoxO1-wt (**A**)**,** control (**C**)**,** and C2C12-FoxO1-AAA (**D**) cells at proliferating (PMB), confluent (CMB), and myotube (MT) stages were detected with immunofluorescence microscopy. The relative distribution of FoxO1 in C2C12-FoxO1-wt cells at these stages was shown in (**B**). The signals of FoxO1 and MHC were detected with Alexa fluor 488 (green) and 486 (red), respectively, conjugated secondary antibody, and nucleus was stained with DAPI (blue). Arrows at the bottom panels point to myotubes. ud: undectable. The original magnification is 200X. (**E**) Vectors expressing GFP-FoxO1-wt (top panel) or GFP-FoxO1-AAA (bottom panel) were transfected into parental C2C12 cells of myotube stage (4 days in DM) and the GFP signal in multinucleated myotubes (detected by MHC antibody, red) was viewed 24 hr after transfection. (**F**)**,** and (**G**)**,** nuclear shuttling of FoxO1 is suspended in myotubes. Proliferating myoblasts (**F**) and differentiated myotubes (**G**) of C2C12-FoxO1-wt were treated with vehicle or leptomycin B (LPB, 2 ng/ml) for 24 h before fixed and stained for FoxO1 and MHC as described above. The original images of (E), (F) and (G) were taken at 400X magnification.

The nuclear localization of FoxO1 is regulated by several signaling pathways and its exclusion from the nucleus prevents its transcriptional regulatory roles on target genes [35]–[37]. Therefore, understanding the subcellular localization of FoxO1 during myogenic differentiation will help reveal its function in this process. Using immunofluorescence assay, we observed that FoxO1-wt could be seen in both cytoplasm and nucleus in proliferating myoblasts (PMB), although slight enrichment in the nucleus could be observed (Fig. 2A & B). Nuclear enrichment was further enhanced in confluent myoblasts (CMB) when cells were abutting each other, suggesting that cell-cell contact signals may enhance the nuclear localization of FoxO1-wt. After 4 days in differentiation medium, most mononucleated cells retained FoxO1 in their nuclei (Fig. 2A & B). However, FoxO1-wt was excluded from nucleus when cells fused to form multinucleated myotubes (Fig. 2A bottom panels & 2B right panel). Similar subcellular localization pattern of endogenous FoxO1 was also observed in control cells (Fig. 2C). Since the subcellular localization patterns of ectopic and endogenous FoxO1 are the same, it suggests that ectopic FoxO1-wt functions similarly to its endogenous counterpart. These observations further suggest that nuclear exclusion of FoxO1 is an important step for terminal myogenic differentiation. This hypothesis was further confirmed when GFP-FoxO1-wt was transfected into myoblasts and similar results were observed (Fig. 2E and Fig. S1). Interestingly, although a complete cytoplasmic localization of GFP-FoxO1 was observed in multinucleated myotubes (Fig. 2E, top panel; N = 35), GFP-FoxO1-wt in mononucleated myoblasts kept in DM for 4 days localized to the nucleus (Fig. S1B), confirming the nuclear exclusion of FoxO1 in multinucleated myotubes.

It is of interest to verify if the sub-cellular localization of FoxO1-AAA is different from that of wildtype FoxO1. At PMB and CMB stages, FoxO1-AAA was strictly in the nucleus (Fig. 2D). Cytoplasmic localization of FoxO1-AAA was only seen in fully differentiated multinucleated myotubes but still with sufficient ratio of FoxO1-AAA still present in the nucleus (Fig. 2D and top 2 panels in Fig. S2). In some rare well differentiated myotubes, FoxO1-AAA was completely in the cytoplasm (Fig. S2, bottom panel). GFP-Foxo1-AAA transfected into myotubes also show nuclear localization (Fig. 2E, bottom panel). These observations confirmed the preferred nuclear localization of FoxO1-AAA as reported before and indicate that signals of terminal differentiation can drive FoxO1-AAA out of nucleus, although with poor efficiency, even when the AKT-targeted sites are mutated.

### Nuclear Import of FoxO1 is Suspended in Myotubes

The cytoplasmic localization of FoxO1 in myotubes suggests that either its nuclear import is prevented/reduced or its nuclear export is enhanced. To find out the actual mechanism, we treated the proliferating myoblasts and differentiated myotubes with leptomycin B, an inhibitor of exportins that blocks most nuclear export activity. FoxO1 in proliferating myoblasts localized majorly to cytoplasm but become mostly nuclear after myoblasts were treated with leptomycin B (Fig. 2F), demonstrating the negative effect of leptomycin B on its nuclear export. Interestingly, the localization of FoxO1 in myotubes was not affected by leptomycin B treatment and most, if not all, FoxO1 was still found in the cytoplasm (Fig. 2G). As leptomycin B effectively blocks its nuclear export in proliferating myoblasts, the absence of FoxO1 in nucleus of leptomycin B treated myotubes suggests that, in myotubes, FoxO1 is not transported into nucleus after its translation in the cytoplasm.

### Insulin and LiCl Synergistically Rescue FoxO1 Inhibited Myogenic Differentiation

**Figure 3:**
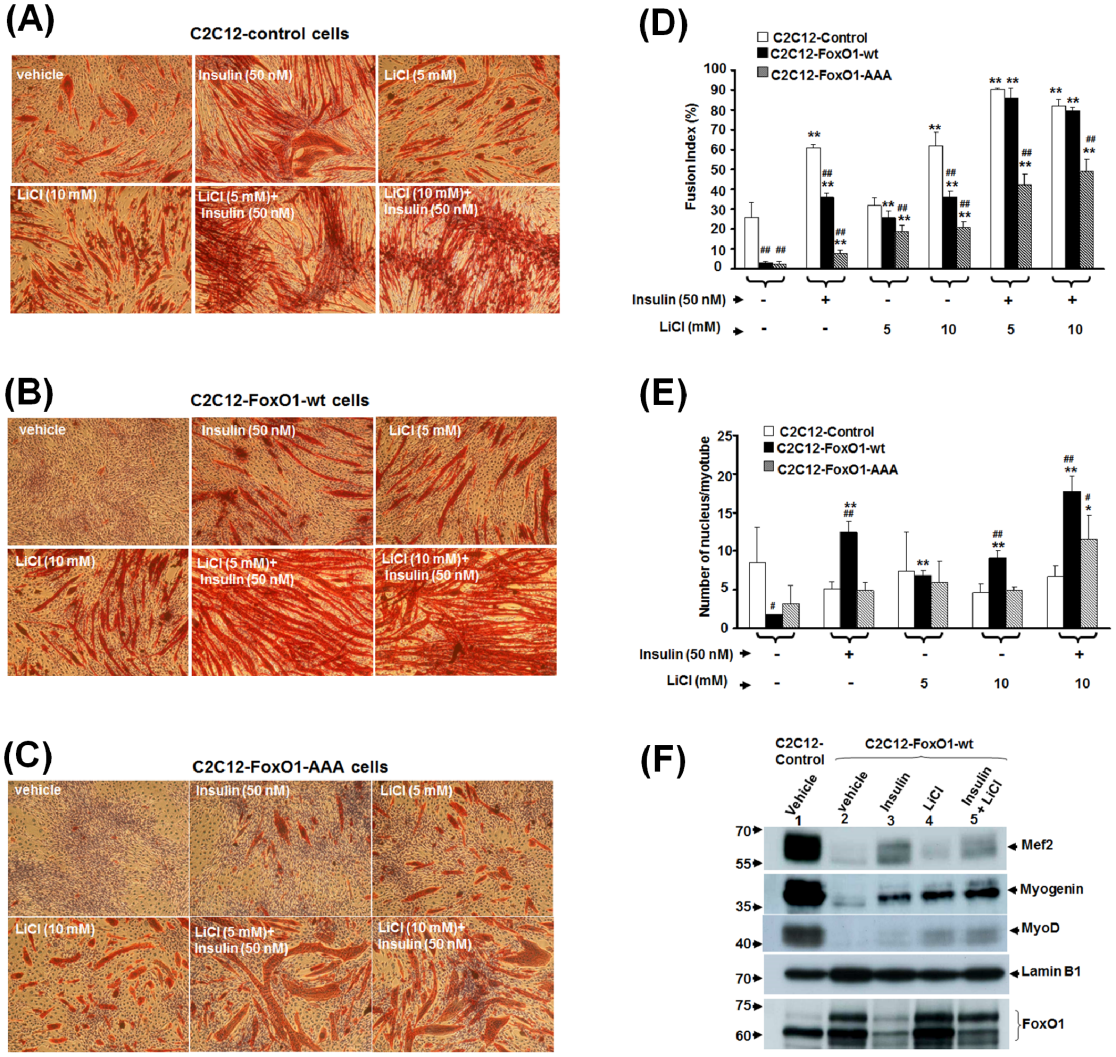
LiCl and insulin synergistically rescue the terminal differentiation of FoxO1 over-expressed stable clones. *Control and FoxO1* over-expressed cells were induced to differentiate in the absence or presence of insulin (50 nM) and/or LiCl (5 mM or 10 mM) for 96 hr. Then, cells were fixed and the signal of MHC (red) was viewed with immunocytochemistry. The morphology of control and FoxO1 (wt or –AAA) over-expressed cells under different treatments was shown in (**A**)**,** (**B**)**,** and (**C**)**,** respectively, and their fusion index and number of nucleus per myotube under various treatments were shown in (**D**) and (**E**)**,** respectively. * and **: p<0.05 and p<0.01 respectively as compared with that of cells treated with vehicle only; # and ##: p<0.05 and p<0.01 respectively as compared to control cells under the same treatment. (**F**)**.** Nuclear level of key myogenic factors in *Control* and *C2C12-FoxO1-wt* cells treated with insulin (50 nM) and/or LiCl (10 mM) for 4 days was detected with Western blot. The signal of Lamin B1 serves as protein input (15 µg nuclear protein) control.

Insulin and IGF-I pathway had been demonstrated as a strong potentiator of myogenic differentiation [38]–[40] and as a critical regulator of FoxOs’ subcellular localization [37]; therefore, it prompted us to examine whether insulin could rescue the differentiation of C2C12-*FoxO1* cells. As would be expected from its nuclear exclusion effect on FoxO1, insulin treatment significantly enhanced and restored the myogenic differentiation of both control and C2C12-FoxO1-wt cells (Fig. 3A & B). The differentiation of C2C12-FoxO1-AAA was also slightly enhanced, although still substantially poorer than that of control and C2C12-FoxO1-wt cells (Fig. 3C), demonstrating that, in addition to nuclear exclusion effect, insulin may regulate FoxO1 activity by other means. Since insulin could only partially rescue the differentiation of C2C12-FoxO1-wt myoblasts (Fig. 3D), it suggested that other activation of other myogenic signaling pathways might also be required to fully rescued the myogenic differentiation of *FoxO1* over-expressed cells. Of special interest was the Wnt signaling pathway that had been shown to play critical role in muscle development [41], [42] and cooperate with insulin to promote myogenesis [43]. Furthermore, the downstream effecter of Wnt signal, β-Catenin, had been found to modulate FoxO transcriptional activity [44]. However, whether these two pathways could collaborate to rescue the myogenic differentiation of C2C12-*FoxO1* cells had not been examined. Here we found that treatment with LiCl, a non-selective inhibitor of GSK3β and potent stimulator of myogenesis [45], significantly enhanced the myogenic differentiation of both control and C2C12-*FoxO1-wt* cells in a dose-dependent manner (Fig. 3A, B, & C). Simultaneous treatment with LiCl and insulin increased the fusion index to about 90% in both control and C2C12-*FoxO1-wt* cells, suggesting that these two treatments promote myogenesis synergistically and can overcome the blocking/inhibitory effect caused by *FoxO1-wt* over-expression. The differentiation of C2C12-FoxO1-AAA in the presence of insulin and LiCl was still significantly lower than that of control and C2C12-FoxO1-wt cells (Fig. 3C & D), confirming again the importance of FoxO1 nuclear exclusion for myogenic differentiation. Closer examination of the myotubes morphology under LiCl plus insulin treatment found that (1) myotubes of the control cells aggregated to form conglomerates without fusing into larger tubes, (2) the size/diameter of myotubes formed by C2C12-*FoxO1* cells, was much larger than that of control cells (Fig. 3E). These observations suggest that this treatment (LiCl+insulin) has turned FxO1 from a myogenic repressor into a factor promoting muscular hypertrophy.

To further explore the effects of insulin and LiCl on myogenesis, we examined the nuclear level of key myogenic factors. Insulin significantly increased nuclear accumulation of Mef2 and Myogenin, while nuclear level of MyoD and Myogenin was enhanced by LiCl (Fig. 3F). The nuclear level of these 3 factors was all increased when cells were treated with insulin and LiCl at the same time, demonstrating that these two treatments act through different pathways and synergize to rescue FoxO1 repressed myogenic differentiation. Nuclear level of FoxO1 was dramatically reduced by insulin but not by LiCl, which suggests that either nuclear exclusion or degradation of FoxO1 is triggered by insulin to reverse FoxO1 inhibited myogenesis but none of these scenarios is induced by LiCl.

### Insulin and LiCl have Different Effects on FoxO1 Subcellular Localization

**Figure 4:**
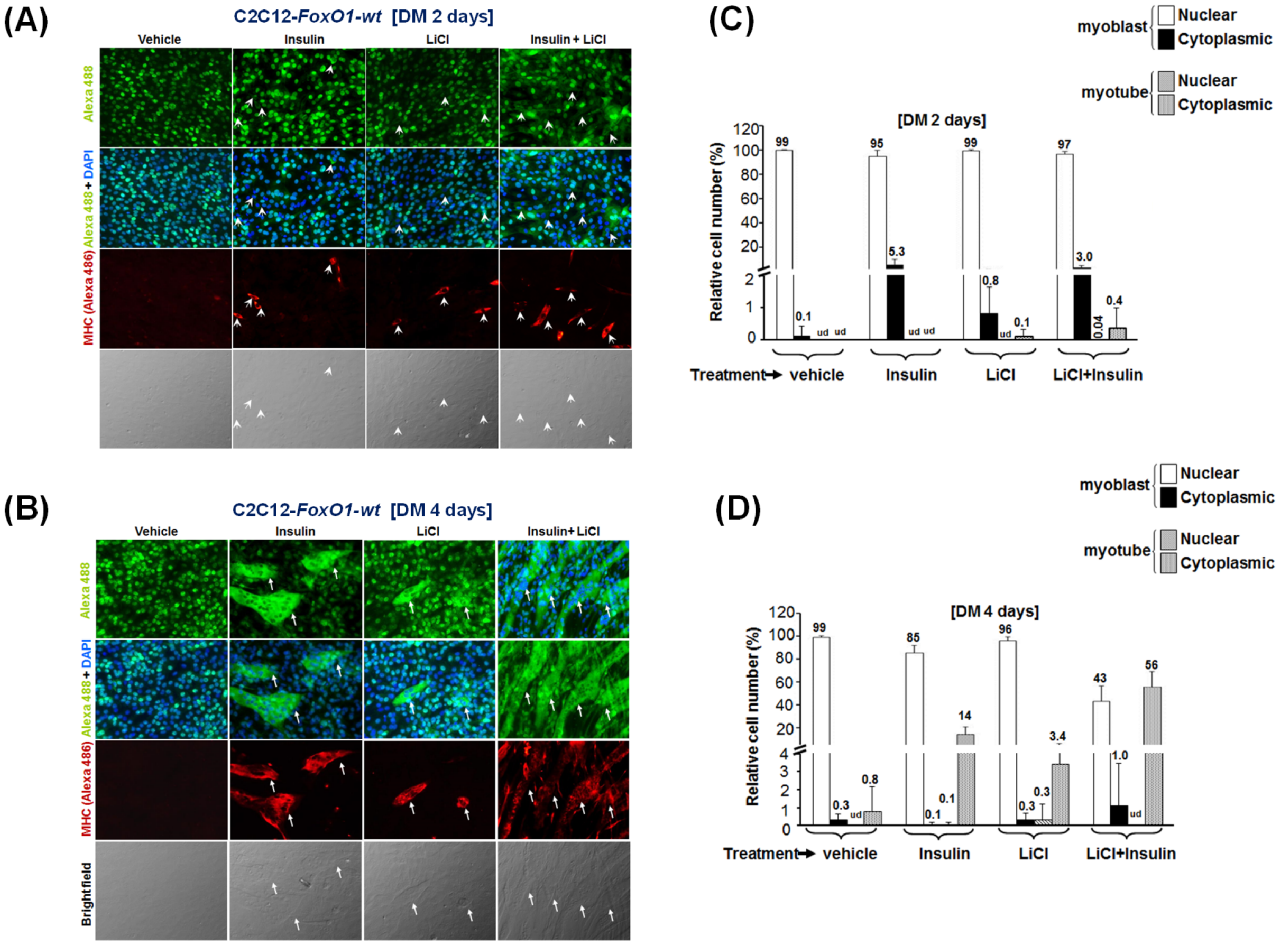
The effects of insulin and LiCl treatments on FoxO1 subcellular localization. The subcellular localization of FoxO1 in C2C12-FoxO1-wt cells treated with/without insulin and LiCl for 2 and 4 days was detected with immunofluorescence microscopy as described in Fig. 2 and results are shown in (**A**) and (**B**) respectively. The relative percentages of myoblasts and multinucleated myotubes with either nuclear or cytoplasmic FoxO1 localization under various treatments were shown in (C) and (D). Arrow heads and arrows indicate MHC-positive myoblasts (mononucleate) and multinucleated myotubes, respectively. The original images were taken at 200X magnification. ud: undectable.

**Figure 5:**
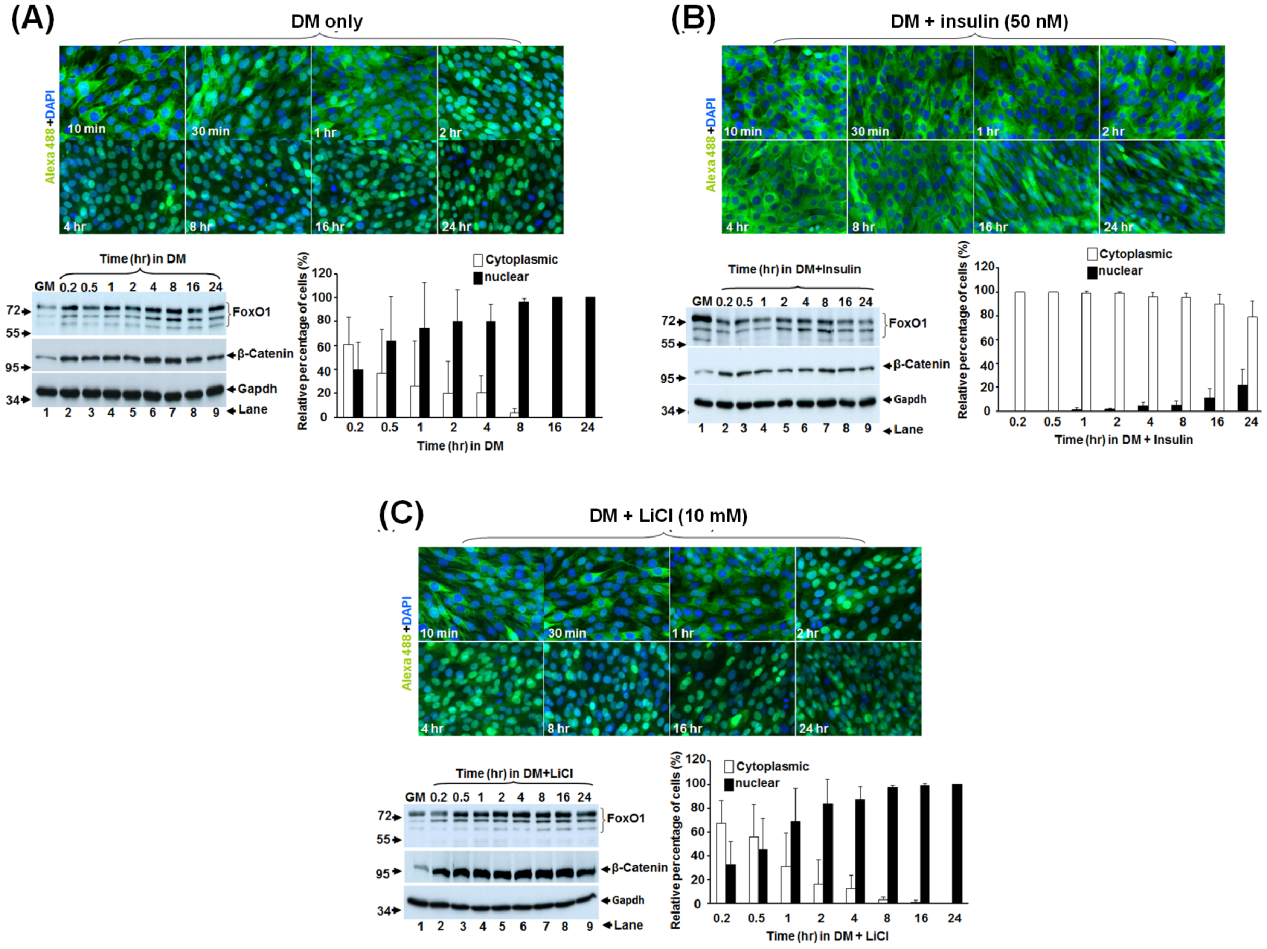
Insulin delays the nuclear accumulation of FoxO1. (**A**) The subcellular localization and expression level of FoxO1 in C2C12-FoxO1-wt cells at various time points after replacement of GM with DM was examined with immunofluorescence microscopy and Western blot, respectively, as described in Fig. 2. The relative percentages of myoblasts with either nuclear or cytoplasmic FoxO1 localization at various time points were shown in the bottom right panel. The expression levels of FoxO1 and β-Catenin in the total lysate (50 µg) at these time points are shown in the bottom left panel and the signal of Gapdh serves as input control. Similar experiments were done with DM containing either insulin (**B**) or LiCl (**C**).

To reveal how insulin and LiCl can promote myogenesis, we started to examine whether they do so by excluding FoxO1 from the nucleus. In C2C12-FoxO1-wt cells left in DM for 2 days (DM2), regardless of the treatments they received, most FoxO1 was still in the nucleus. Among these treatments, only less than 1% of cells treated with insulin plus LiCl had formed multinucleated myotubes and excluded their FoxO1 to the cytoplasm (Fig. 4A & C). Further incubation in DM for another 2 days (DM4) enhanced myotube formation in all treatments (Fig. 4B), and synergistic effect of insulin and LiCl on myotube formation was also observed. As in differentiated control cells (Fig. 2), FoxO1 was excluded from the nucleus of all multinucleated myotubes but most FoxO1 in mononucleated myoblasts was still confined in the nucleus, except in some MHC-positive ones (Fig. 4B & D). Although these observations confirmed again the importance of nuclear exclusion of FoxO1 in myotube formation, it failed to answer whether insulin and LiCl act by altering subcellular localization of FoxO1. To better dissect the effect of these treatments on FoxO1 shuttling, a time course tracing of FoxO1 with shorter intervals after treatments was performed. As described above, most FoxO1 was in the nucleus in confluent myoblasts grown in GM (Fig. S3); however, to our surprise, replacement of the GM by DM alone had shifted FoxO1 from nucleus to cytoplasm in 10 min (Fig. 5A and Fig. S3), and the total amount of both FoxO1 and β-Catenin was significantly increased in DM in less than 10 min and sustained at this level for at least 24 hr (Fig. 5A, bottom left panel). Nuclear localization of FoxO1 level started to recover in 2 hr and returned to the nucleus of more than 95% of cells in 8 hr (Fig. 5A, bottom right panel). Upon this GM-to-DM induced shuttling pattern, we observed only 34% of cells with nuclear FoxO1 after 24 hr in insulin, implying that insulin delayed its return to nucleus (Fig. 5B). Apart from delaying its nuclear localization, insulin also significantly reduced the protein level of FoxO1 (Fig. 5B, bottom left panel). Since the expression of ectopic FoxO1 was driven by a constitutively active promoter (retroviral LTR promoter), it suggests that insulin reduces the stability of FoxO1 protein. Treatment with LiCl had no significant effect on the nucleus-cytoplasm shuttling pattern and the FoxO1 protein level (Fig. 5C), implying that LiCl may promote myogenesis through other means.

### FoxO1 Directly Repressed the Promoter Activity of Myogenic Genes

**Figure 6:**
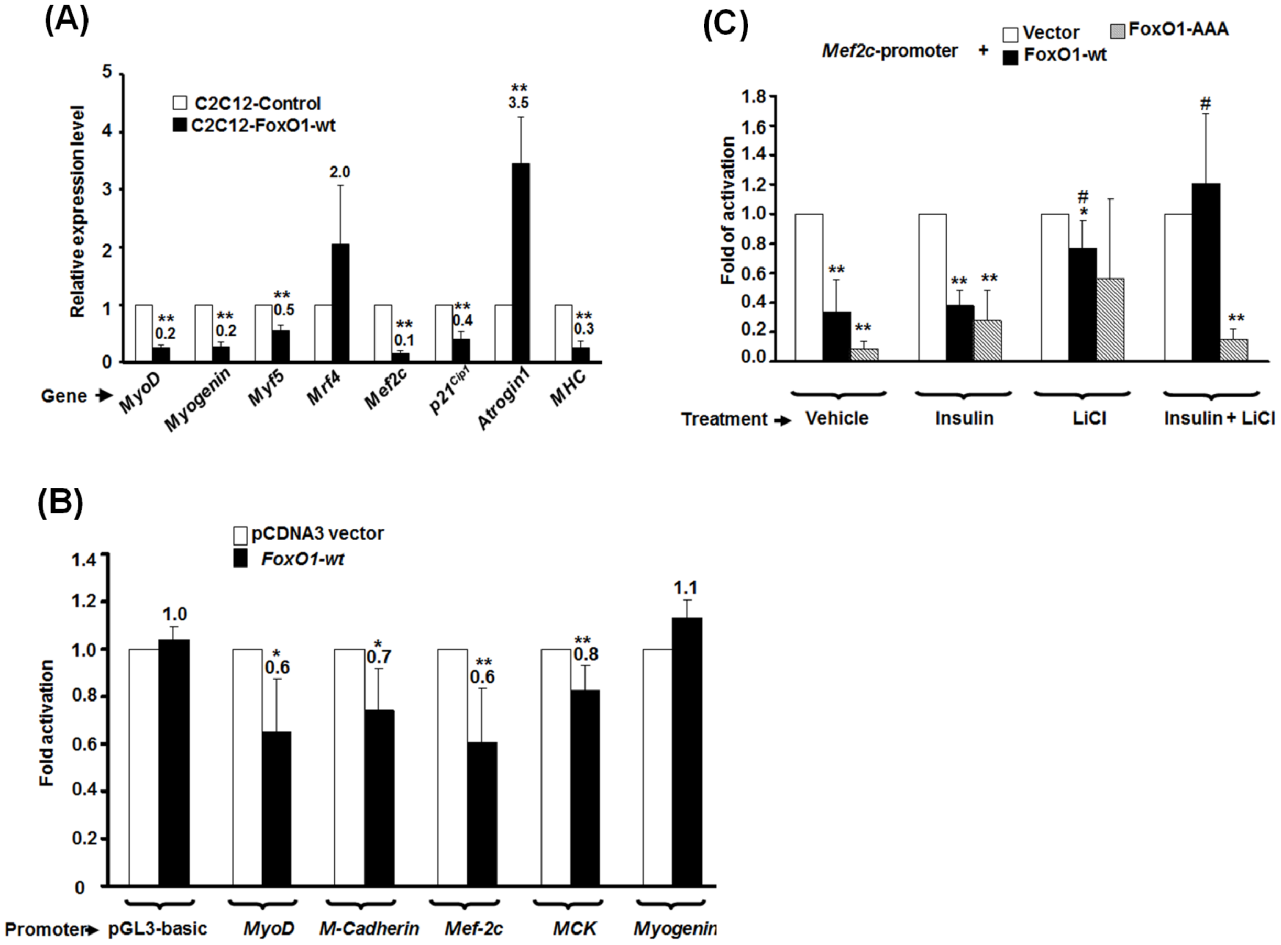
FoxO1 represses the promoter activity of myogenic genes. (**A**) The expression level of myogenic genes in *Control* and *C2C12-FoxO1-wt* stable clones of MT stage was determined by quantitative real time RT-PCR. The expression level of each gene was normalized to that of *Gapdh* (ΔCt ). Normalized expression level of each gene in C2C12-FoxO1-wt cells was compared with that of control cells and the ratio (2^−ΔΔCt^) is shown here. Results shown are means ± SD of two independent experiments. * and **: p<0.05 and p<0.01 respectively as compared with that of control cells. (**B**) Myogenic gene promoters driven reporters were co-transfected with pCDNA3-*FoxO1* vector into C2C12 to test the effect of *FoxO1* expression on their promoter activity. The luciferase activity of each reporter in the absence of FoxO1 was arbitrarily set as 1 fold activation and their activity in the presence of FoxO1 was compared to that. (**C**) C2C12 cells transfected with *Mef2c* promoter driven reporter and expression vectors of *FoxO1-wt* and *–AAA* were treated with insulin (50 nM) and/or LiCl (10 mM). The luciferase activity of *Mef2c* promoter in the absence of *FoxO1* expression vectors was arbitrarily set as 1 fold activation. Results shown are means and S.D. of at least 3 independent experiments. * and **: p<0.05 and p<0.01 respectively as compared with that of cells transfected with reporter and empty expression vector only. #: p<0.05 as compared with that of cells transfected with reporter and the same expression vector under vehicle treatment.

It was of interest to understand how over-expression of *FoxO1* repressed myogenic differentiation. To answer this question, we set out to analyze the expression pattern of key factors regulating myogenesis, such as *MRFs*, *Mef2c*, and genes of contractile protein, during the process of terminal differentiation (Fig. 6A). The expression of *Atrogin-1,* a muscle atrophy promoter and a well-known target of FoxO1 [20], was also examined. Except for *Mrf4*, the expression of all myogenic genes was reduced in FoxO1 over-expressed cells. In sharp contrast to the reduction of myogenic genes, the expression of *Atrogin-1* was enhanced by FoxO1, as reported by other studies. Taken these results together, it suggests that FoxO1 can inhibit serum withdrawal induced myogenic differentiation by repressing myogenic genes expression and increasing *Atrogen-1* expression.

It was important to know whether FoxO1 repressed the expression of myogenic genes by direct targeting their promoters. Using transient transfection promoter assay, we found that the promoter activities of *MyoD* and its target genes, including *M-cadheirn*, *Mef2c*, and *muscle creatine kinase* (*MCK*), was significantly repressed by FoxO1 in C2C12 cells (Fig. 6B). No repression of *Myogenin* promoter activity by FoxO1 was observed. The effect of LiCl and insulin treatments on *Mef2c* promoter was further examined. Surprisingly, addition of LiCl, but not insulin, partially removed FoxO1 mediated repression (Fig. 6C); however, simultaneous treatment of LiCl and insulin fully rescued *Mef2c* promoter activity. These observations suggest that FoxO1 can directly repress the promoter activity of myogenic genes to block myogenic differentiation and this repression can be completely removed by LiCl and insulin treatment.

## Discussion

### FoxO Isoforms and Terminal Myogenic Differentiation

Although the first FoxO gene in vertebrates was discovered in human rhabdomyosarcoma, the exact roles played by these factors during myogenesis remain largely controversial. They are expressed in pluripotent stem cells and are critical mediators for combating metabolically-derived oxidative stress and maintenance of pluripotency [46], [47]. The phenotype caused by disruption of each FoxO genes is very different, and it suggests that they have some degree of functional diversification during development [48], [49]. However, none of the gene disruption embryos shown significant disturbance in their somitic development, except for smaller size observed in *FoxO1^−/−^* embryos, suggesting that FoxOs play redundant roles during embryonic myogenesis and the results obtained in this study with FoxO1 may be extrapolated to other FoxOs, especially FoxO3 and FoxO4 that have similar expression and subcellular localization pattern during myogenesis. The smaller size caused by disruption of *FoxO1* gene implies that this gene might play an important role in the development of skeleton and musculature and this notion is consistent with our discovery that FoxO1 might, in the presence of Wnt and insulin, actually potentiate myogenesis. The differentiation niche of myoblasts *in vivo* is full of myogenic signaling molecules, and previous conclusions about the inhibitory effect of FoxO1 on myogenesis is probably an artifact caused by serum withdrawal for inducing myoblasts terminal differentiation *in vitro*.

### Signaling Pathways Mediating FoxOs’ Effects on Myogenic Differentiation

Several signaling pathways, such as Notch, mTOR, and Myostatin, have been reported to mediate the repressive effect of FoxOs on myogenic differentiation [50]–[52]. FoxO1 interacts with Notch intracellular domain on the promoter of *Csl*, a Notch downstream effector, leading to the activation of Notch target genes. The FoxO1 repressed myogenesis can be partially rescued by inhibition of Notch signaling [51]. Direct activation of the promoters of atrophy-promoting genes, including *Myostatin, Atrogin-1*, and *MuRF1*, by FoxOs has been observed [52], and this lead to the degradation of a subset of components of the mTOR signaling network and consequent prevention of myogeneis. In view of the strong repressive effect on myogenesis, it has long been speculated that FoxO1 should have a direct regulatory role on myogenesis, instead of acting through signaling pathways indirectly. Here we found that the expression of most MRF, except for *Mrf4*, was reduced by FoxO1 and direct repression of *MyoD* and its target genes’ promoters by FoxO1 was also demonstrated (Fig. 6). We were surprised to find that FoxO1 failed to repress the 1.6 kb *Myogenin* promoter that had been shown to harbor most, if not all, essential regulatory elements of *Myogenin* gene. Thus, the repression of *Myogenin* expression found here might be achieved either by distal FoxO1-binding sites or through indirect pathways, such as reduction of *MyoD* expression. Taken together, these observations suggest that FoxO1 can repress serum withdrawal induced myogenic differentiation through both direct and indirect pathways. In the future, we will further define the FoxO1-binding sites in these promoters and examine their occupancy by FoxO1 during myogenesis *in vitro* and *in vivo*.

### Insulin Regulates FoxO1 Activity through Multiple Mechanisms

PI3K-Akt pathway is one of the major pathways mediating insulin signal received on the plasma membrane. Therefore, the downstream targets of Akt, including FoxOs, are the major effectors of insulin signaling in various organs [53]. In addition to FoxOs, Akt phosphorylates a wide range of factors, including mTOR, GSK-3β, and AS160. Activation of Akt/mTOR pathway and inhibition of GSK-3β has been found to promote muscle hypertrophy [54]. Therefore, treatment with insulin may have pleotropic effects due to the activation of the downstream effectors of several signaling pathways. The most obvious effect of insulin on FoxO1 has been attributed to its nuclear exclusion, which is also observed in this study. However, this observation does not exclude the possibility that insulin can also regulate FoxO1 activity by other means at the same time. This speculation has got strong support from previous findings that (1) insulin signaling can inhibit the FoxO-mediated target gene transactivation even their NES signal was destroyed [55], and (2) the phosphrylation of FoxOs is increased during terminal differentiation and most of them are still in the nucleus of mononucleated myoblasts [22], [26]. Here we have observed strong reduction of FoxO1 level upon insulin treatment (Fig. 5B), demonstrating that insulin can also regulate FoxO1 activity by changing its protein stability as reported in other cell types [56]–[58]. The degradation of FoxO1 in other cells was shown to be mediated by the 26S-proteosome system and phosphorylation of the Akt target sites was necessary for ubiquitination [57], [58].

It was intriguing to find that insulin promoted FoxO1 nuclear exit and degradation (Fig. 5B) while FoxO1 potentiated myogenesis upon insulin treatment (Fig. 3D & E). Although the mechanism is currently uncertain, it can be achieved through a few possible pathways. One possibility is that over-expression of the FxO1 in the cells changed the metabolic sensitivity to the assimilative effect of insulin so cells become more hypertrophic after insulin treatment [59]. Alternatively, cytoplasmic FoxO1 may exert non-genomic effect by binding to factors involved in signal transduction or metabolism to potentiate myogenesis. For instance, autophagy has been implicated in myogenic differentiation and glucose homeostasis [60]; in the meantime, cytosolic FoxO1 has been demonstrated as an important mediator of autophagy [61]. Therefore, in insulin treated cells, residual cytoplasmic FoxO1 may potentiate myogenesis via regulating autophagy. It will be interesting to test if these mechanisms mediate the myogenesis-potentiating activity of FoxO1 upon insulin treatment.

### LiCl may Activate β-Catenin and other Factors to Recue FoxO1 Inhibited Myogenesis

Wnt signaling pathway has been found to play critical role in muscle development [41], [42] and cooperates with insulin to promote myogenesis [43], but it is unknown whether LiCl or activation of Wnt signaling pathway can rescue FoxO1 repressed myogenesis. It has been shown that β–Catenin can promote myogenesis by interacting with MyoD and enhances its binding to E box elements and transcriptional activity; besides, the transactivation of MyoD is inhibited when β-catenin is either deficient or the interaction between MyoD and β–catenin is prevented [41]. Additionally, β-catenin can also enhance myogenesis by relieving I-mfa-mediated suppression of myogenic regulatory factors [62]. Therefore, activation of canocal Wnt signaling by LiCl may rescue myogenesis at multiple levels. We also found that the rescuing effect of LiCl was synergistic with insulin treatment (Fig. 3D). Since both Wnt and insulin signals inhibit GSK 3β and thus facilitate the accumulation of β-Catenin in the cytoplasm [63], the synergism between insulin and LiCl treatments implies that other additional mechanisms must also be employed by them to promote and rescue FoxO1 blocked myogeneis. We found that insulin reduced FoxO1 protein level and delayed its return to nucleus, but similar effect was not observed with LiCl (Fig. 3E and 5). Moreover, the FoxO1 mediated repression of myogenic gene promoters can be partially relieved by LiCl but not by insulin alone (Fig. 6C), implying that residual nuclear FoxO1 after insulin treatment is enough to repress *Mef2c* promoter, and the synergism between GSK3β/β-Catenin pathway and other pathways induced by LiCl treatment should play critical role in preventing the repression of myogenic gene promoters by FoxO1. Simultaneous treatment with insulin and LiCL activated all these pathways together and at the same time reduced the nuclear level of FoxO1; therefore this treatment successfully rescued myogenic gene promoter activity and myogenesis.

It was a surprise to find that LiCl and insulin co-treatment did not rescue FoxO1-AAA repressed *Mef2c* promoter activity (Fig. 6C), since the same treatment significantly rescued FoxO1-AAA repressed myogenesis (Fig. 3C & D). This observation suggests that different myogenic genes might respond differentially to insulin and LiCl treatment as evidenced in the response of *Mef2*, *Myogenin*, and *MyoD* expression to these treatments (Fig. 3F). *Mef2c* promoter might be more sensitive to the presence of FoxO1-AAA than other myogenic genes and thus less responsive to the rescuing effect of LiCl and insulin. The observed myogenesis rescue might be attributed collectively to the activation of some myogenic genes, such as *Myogenin*, that are highly responsive to LiCl and insulin.

It will be an interesting task to identify the unknown pathways employed by LiCl to rescue FoxO1 repressed myogenesis. One important myogenic signaling pathway targeted by LiCl is p38 MAPK. Lithium increases p38 MAPK activity and stimulate glucose uptake regardless of the status of insulin [64]. Activation of p38 MAPK promotes myogenic differentiation and rescues the differentiation of rhabdomyosarcoma by enhancing the transcriptional activity of both MRFs and MEF2 families [65], [66]. Therefore, in the future, more endeavors are required to elucidate whether activation of p38 MAPK is mediating the rescuing effect of LiCl on FoxO1-wt inhibited myogenic gene, especially *Mef2c*, expression during myogenesis.

## Supporting Information

Figure S1 **Subcellular localization of GFP-FoxO1-wt in C2C12 myoblasts and myotubes.** Parental C2C12 cells of PMB **(A)** and myotube **(B)** stages were transfected with GFP-FoxO1-wt expressing vector and the GFP-FoxO1-wt signal was viewed 24 hr after transfection to reveal their localization in mononucleated myoblasts. GFP expressing vector was also transfected into multinucleated myotubes (detected by MHC antibody, red) as above to serve as a control (B, bottom panel). Two representative images are shown in **(A)**. Arrows indicate transfected cells.
(TIF)

Figure S2 **Subcellular localization of FoxO1-AAA in C2C12-FoxO1-AAA cells of DM4 stage.** C2C12-FoxO1-AAA cells were kept in DM for 4 days and harvested for detecting the subcellular localization of FoxO1-AAA and MHC as described in Fig. 2. Arrows indicate multinucleated myotubes.
(TIF)

Figure S3 **Subcellular localization of FoxO1-wt shortly after differentiation.** The localization of FoxO1-wt in C2C12-FoxO1-wt cells shortly (0, 5, and 10 min) after the replacement of GM by DM with/without insulin (50 nM) or LiCl (5 mM) was detected with immunofluorescence microscopy as described above. The original images were taken at 400X magnification.
(TIF)
