# Supplementary figures and images for: Insulin and LiCl Synergistically Rescue Myogenic Differentiation of FoxO1 Over-Expressed Myoblasts
Source: PLoS One. 2014 Feb 13;9(2):e88450. doi: 10.1371/journal.pone.0088450 (PMC3923792; doi:10.1371/journal.pone.0088450)

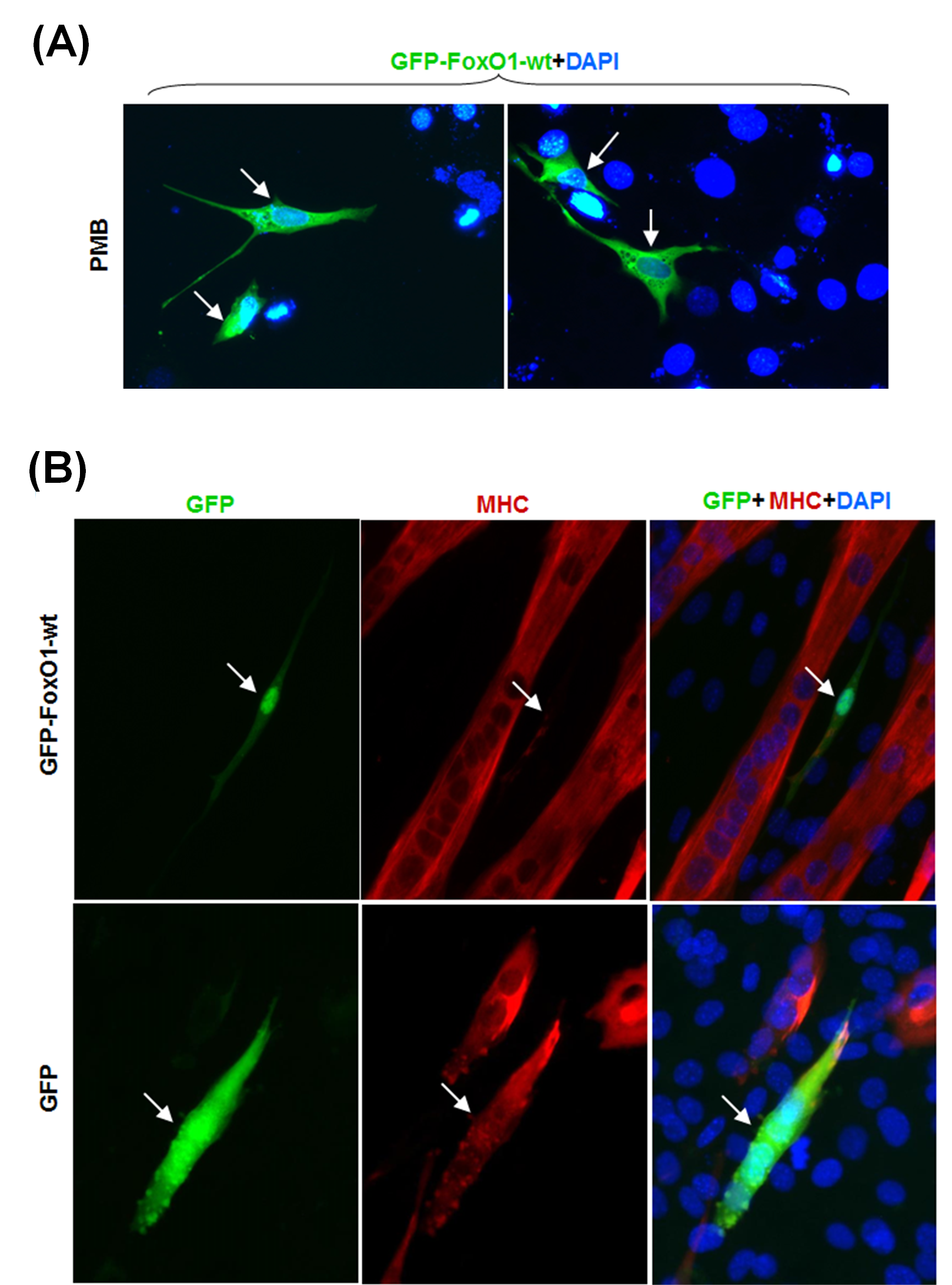

Supplement: Figure S1 — Subcellular localization of GFP-FoxO1-wt in C2C12 myoblasts and myotubes. Parental C2C12 cells of PMB (A) and myotube (B) stages were transfected with GFP-FoxO1-wt expressing vector and the GFP-FoxO1-wt signal was viewed 24 hr after transfection to reveal their localization in mononucleated myoblasts. GFP expressing vector was also transfected into multinucleated myotubes (detected by MHC antibody, red) as above to serve as a control (B, bottom panel). Two representative images are shown in (A). Arrows indicate transfected cells. (TIF) [file pone.0088450.s001.tif]

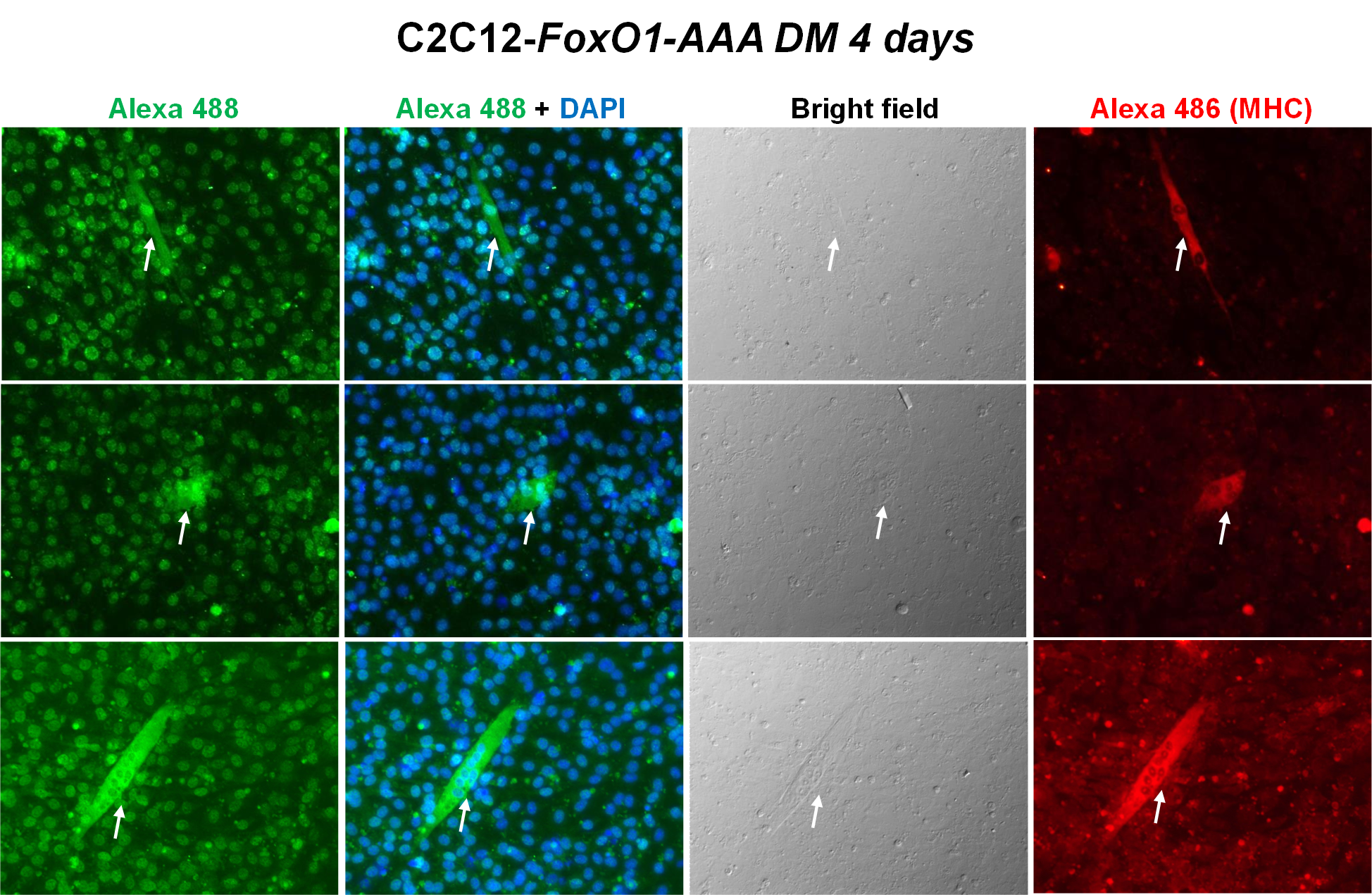

Supplement: Figure S2 — Subcellular localization of FoxO1-AAA in C2C12-FoxO1-AAA cells of DM4 stage. C2C12-FoxO1-AAA cells were kept in DM for 4 days and harvested for detecting the subcellular localization of FoxO1-AAA and MHC as described in Fig. 2. Arrows indicate multinucleated myotubes. (TIF) [file pone.0088450.s002.tif]

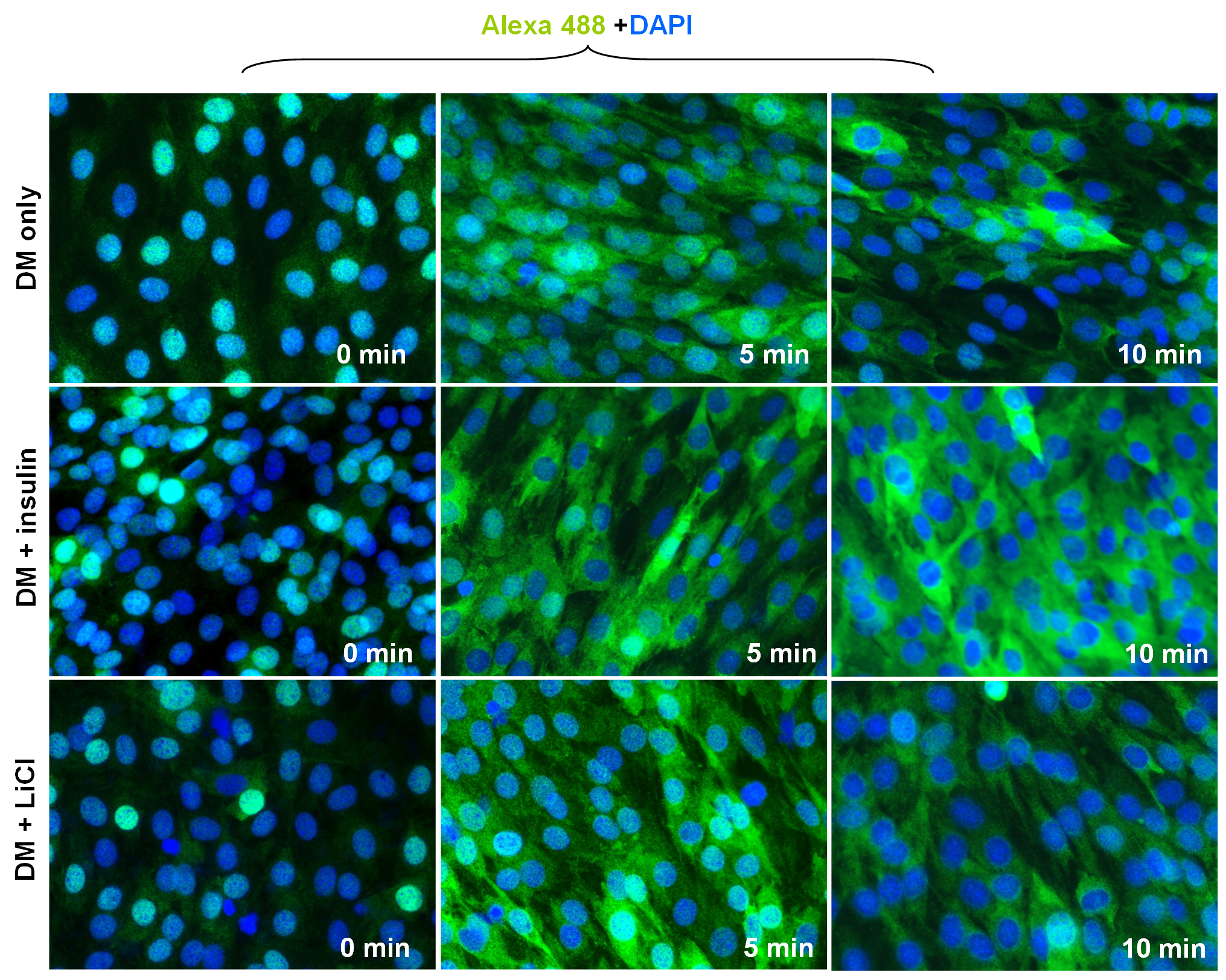

Supplement: Figure S3 — Subcellular localization of FoxO1-wt shortly after differentiation. The localization of FoxO1-wt in C2C12-FoxO1-wt cells shortly (0, 5, and 10 min) after the replacement of GM by DM with/without insulin (50 nM) or LiCl (5 mM) was detected with immunofluorescence microscopy as described above. The original images were taken at 400X magnification. (TIF) [file pone.0088450.s003.tif]
